# Successful Treatment of Disseminated *Acanthamoeba* sp. Infection with Miltefosine

**DOI:** 10.3201/eid1411.070854

**Published:** 2008-11

**Authors:** Alexander C. Aichelburg, Julia Walochnik, Ojan Assadian, Helmut Prosch, Andrea Steuer, Gedeon Perneczky, Govinda S. Visvesvara, Horst Aspöck, Norbert Vetter

**Affiliations:** Otto Wagner Hospital, Vienna, Austria (A.C. Aichelburg, A. Steuer, N. Vetter, H. Prosch); Medical University of Vienna, Vienna (J. Walochnik, O. Assadian, H. Aspöck); Krankenanstalt Rudolfstiftung, Vienna (G. Perneczky); Centers for Disease Control and Prevention, Atlanta, Georgia, USA (G.S. Visvesvara)

**Keywords:** Acanthamoeba, granulomatous Acanthamoeba encephalitis (GAE), tuberculosis, immunocompromised non–HIV-infected patient, miltefosine, dispatch

## Abstract

We report on an HIV-negative but immunocompromised patient with disseminated acanthamoebiasis, granulomatous amoebic encephalitis, and underlying miliary tuberculosis and tuberculous meningitis. The patient responded favorably to treatment with miltefosine, an alkylphosphocholine. The patient remained well with no signs of infection 2 years after treatment cessation.

A 25-year-old man from India, who had been living in Austria for 7 years and had no previous history of major illnesses, was brought by ambulance to the hospital for dyspnea, cough, fever, and weight loss. During neurologic examination, a hearing impairment was suspected. The patient was unable to walk because of severe ataxia. Skin examination showed several necrotic ulcers with purulent discharge and black eschars, measuring 0.5 cm to 3 cm, located on the skull, back, neck, and arms (Figure 1, panels A and B). Miliary tuberculosis (TB) of the lungs, liver, spleen, and kidneys was suspected on the basis of chest radiography and computed tomography (CT) of chest and abdomen. Ziehl-Nielsen (ZN) staining for acid-fast bacilli in sputum, bronchial secretions, and lavage obtained through bronchoscopy was negative. PCR for *Mycobacterium tuberculosis* in bronchial secretions and serum was positive. Culture on Loewenstein agar resulted in growth of nonresistant *M. tuberculosis* after 31 days. Blood cultures were negative for aerobic/anaerobic bacteria, mycobacteria, and fungi. Results of serologic tests were negative for *Aspergillus, Candida, Cryptococcus, Histoplasma, Blastomyces,* and *Coccidioides* spp. Severe immunosuppression with a CD4+ lymphocyte count of 182 cells/μL made HIV infection probable, but HIV testing results were negative. Cranial CT showed multiple small enhancing lesions in cerebral cortex and underlying white matter, pons, midbrain, and around most of the cisterns. On magnetic resonance imaging (MRI), the lesions appeared as high T2 signal areas that enhanced heterogeneously or in a ringlike manner. These findings were compatible with the diagnosis of meningoencephalitis with intracerebellar abscess formation.

**Figure 1:**
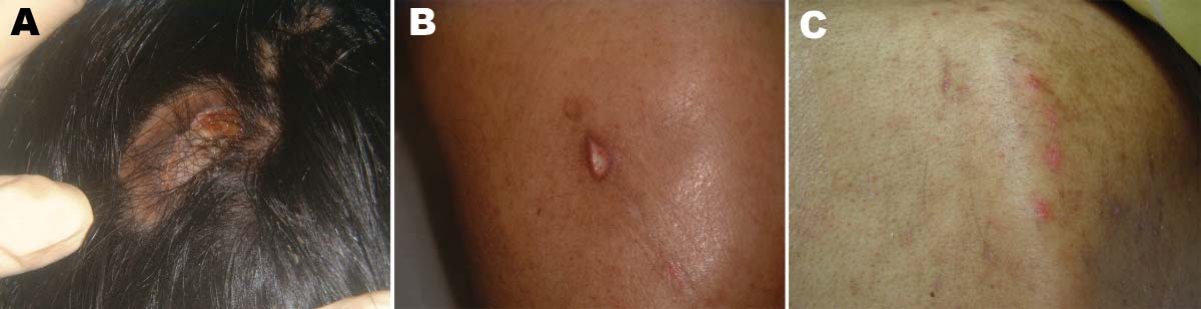
Ulcer with purulent discharge located on the skull (A) and on the back at first examination (B) and 17 days after topical treatment with miltefosine was initiated (C).

Cerebrospinal fluid (CSF) obtained through lumbar puncture was negative for *Toxoplasma gondii, Encephalitozoon cuniculi,* and *Enterocytozoon bieneusi* by PCR and for *Trypanosoma gambiense* by indirect hemagglutination assay. Staining and antigen testing (enzyme immunoassay) for *Cryptococcus neoformans* was negative, as was *Treponema pallidum* antibody testing. No viruses (herpes simplex 1 and 2, varicella zoster, enterovirus) could be detected by PCR. Cultures were negative for aerobic/anaerobic bacteria and fungi. ZN staining detected acid-fast bacilli that were confirmed to be nonresistant *M. tuberculosis* after culture for 38 days. PCR for *M. tuberculosis* was positive. An *Acanthamoeba*–specific PCR (*1*) and DNA sequencing of the PCR product showed *Acanthamoeba* genotype T2 (corresponding to group III). High immunoglobulin (Ig) G (2,000) and IgM (1,000) titers against *Acanthamoeba* spp. could be demonstrated serologically. The organism could not be grown in culture (*2*).

Two skin-biopsy specimens were obtained; they showed necrotizing granulomatous inflammation affecting the entire dermal thickness and subcutis. Stains and culture were negative for *Mycobacterium* spp., fungi, and *Acanthamoeba* spp. but tested positive for acanthamoebae by PCR. In addition to a standard tuberculostatic 5-drug regimen including intravenous streptomycin, empiric anti-amoebic treatment based on data retrieved from the few case reports of successful treatment of systemic *Acanthamoeba* infections (*3*–*5*) was initiated. The regimen included a combination of parenteral trimethoprim/sulfamethoxazole (later changed to oral sulfadiazine) and parenteral fluconazole. CSF samples drawn 2 and 8 weeks after initiation of therapy tested negative for mycobacteria by ZN staining, PCR, and culture but remained positive for *Acanthamoeba* spp. by PCR.

Transbronchial lung biopsy specimens from a second bronchoscopy performed 1 month after admission tested negative for mycobacteria by ZN staining, PCR, and culture but positive for *Acanthamoeba* spp. by PCR. Acanthamoebae could not be cultivated from bronchial secretions or biopsy sample; immunostaining that used a polyclonal rabbit anti–*A. castellanii* (genotype T4) serum was negative.

Within 12 weeks after initiation of tuberculostatic therapy, complete clinical and radiologic resolution of miliary TB of lungs, liver, spleen, and kidneys could be achieved, and the CD4+ lymphocyte count increased to 421 cells/μL. Nevertheless, the neurologic status of the patient deteriorated, even after liposomal amphotericin B and flucytosine had been added to the regimen. Consecutive cranial CT and cranial MRI scans demonstrated progression of the lesions, with the biggest lesion (1.8 cm in diameter) located in the right cerebellopontine angle and cerebellum (Figure 2, panels A and B).

**Figure 2:**
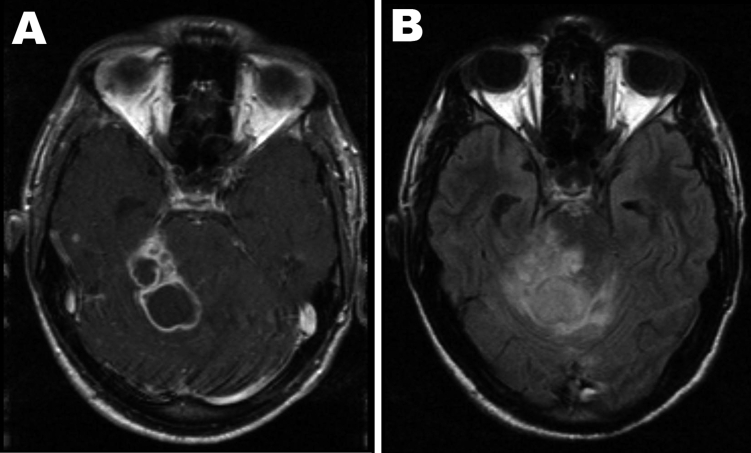
Axial brain magnetic resonance imaging at the level of the cerebellum performed 6 weeks after initial consultation. A) Contrast-enhanced T1 weighted image showing several ring-enhancing lesions in the right cerebellar hemisphere and the right cerebellar peduncle. B) The corresponding fluid attenuation inversion recovery image illustrates the extensive perifocal edema exerting a severe mass effect through compression and displacement of the fourth ventricle with consecutive enlargement of the lateral ventricle.

As skin lesions were also gaining size, treatment with miltefosine, a phosphocholine analog that has proven successful in treating visceral leishmaniasis (*6*) and is highly effective against acanthamoebae in vitro (*7*,*8*), was initiated topically as a solution, 60 mg/mL, 1 drop applied directly to each skin lesion 2 times a day. After dramatic improvements of the skin lesions were observed within only 3 weeks (Figure 1, panel C), our patient began peroral miltefosine 100 mg/day (2.5 mg/kg); all other drugs except the tuberculostatic 5-drug regimen were stopped.

On MRI scan, performed 8 weeks after admission, the brain abscess located in the right cerebellopontine angle had reached a size of 4 cm, leading to a rise of intracranial pressure that could not be controlled by intravenous administration of mannitol and dexamethasone. Nine weeks after admission, an Ommaya Reservoir was implanted, and up to 100 mL of CSF was drained daily to control intracranial pressure. Amikacin, the only other drug that seemed to have some efficacy against *Acanthamoeba* spp. in vitro (*2*), was given intrathecally and intravenously (20 mg/2 mL intrathecally + 1,000 mg/d intravenously) in place of the streptomycin originally included in the 5-drug regimen, under continuous monitoring of peak and trough levels in CSF and blood. A surgical excision of the abscess was performed 3 weeks later. The histologic specimen of this lesion was again positive for *Acanthamoeba* spp. by PCR but negative by immunostaining. Neither acanthamoebae nor mycobacteria could be grown in culture, despite positive ZN staining of the specimen (Table).

**Table:** Diagnosis of *Acanthamoeba* spp. and mycobacterial infection*

Organism, patient specimen	Microscopy	PCR	Culture	Immunostaining	Serology
*Acanthamoeba* spp.					
Cerebrospinal fluid	Negative	Positive	Negative	NA	NA
Brain biopsy	Negative	Positive	Negative	Negative	NA
Bronchial secretions	Negative	Positive	Negative	NA	NA
Lung biopsy	Negative	Positive	Negative	Negative	NA
Skin biopsy	Negative	Positive	Negative	NA	NA
Blood	NA	NA	NA	NA	Positive
*Mycobacterium tuberculosis*					
Cerebrospinal fluid	Positive	Positive	Positive	NA	NA
Brain operative specimen	Positive	Positive	Negative	NA	NA
Sputum	Negative	Positive	NA	NA	NA
Bronchial secretions	Negative	Positive	Positive	NA	NA
Lung biopsy	Negative	Negative	Negative	NA	NA
Blood	NA	Positive	Negative	NA	NA

*NA, not available.

Under ongoing therapy with miltefosine, amikacin, and 4 more tuberculostatic drugs, the patient improved. The remaining cerebral lesions regressed in size. Healing of the dermal lesions was achieved within 6 weeks; topical miltefosine treatment was stopped after 8 weeks. Intrathecal amikacin and oral miltefosine therapy was halted 6 and 12 weeks, respectively, after initiation.

A 2-drug tuberculostatic therapy was maintained for 1 year after the patient had been discharged from hospital. Two more lumbar punctures were performed 23 and 29 weeks after the patient was initially evaluated. For the first time neither mycobacteria nor acanthamoebae could be detected. CT scan and MR imaging of the brain showed no major pathology. Serologic titers, which had gradually declined after initiation of miltefosine therapy, reached normal levels. The patient was transferred to a specialized neurologic institution for rehabilitation. Ataxia and hearing impairment did not improve. During the next 24 months, the patient was regularly seen in our outpatient clinic. No signs of infection could be found, and *Acanthamoeb*a immunoreactivity remained below cutoff.

Disseminated *Acanthamoeba* infection is a rare disease characterized by widespread granulomatous infiltration of the skin and extracerebral organs; it usually occurs in immunocompromised patients. Most reported cases have progressed to granulomatous amoebic encephalitis (GAE). The incidence of GAE is low in spite of the ubiquity of these amoebae. Although <200 cases of GAE have been described worldwide, it is still of substantial medical relevance because it is usually fatal due to diagnostic difficulties (*9*,*10*) and lack of effective treatment.

In our patient, co-infection with *M. tuberculosis* with severe immunosuppression may have contributed to his susceptibility to *Acanthamoeba* infection but not to the disease progression and clinical deterioration seen even after TB could be controlled. Problems in culturing acanthamoebae from clinical specimens have been reported frequently, and isolation of amoebae from CSF is generally uncommon (*11*). In our case no reactivity to immunofluorescence was seen, either because the biopsy missed the area of active infection or because of the low sensitivity of polyclonal antibodies available. Nevertheless, early diagnosis of *Acanthamoeba* infection in our patient was achieved by molecular methods that proved to be more sensitive than microscopy and culture.

GAE and cutaneous *Acanthamoeba* infections have been empirically treated with a wide array of antimicrobial agents. The outcome has been mostly failure (*3*,*11*,*12*), except for a few cases that occurred in immunocompetent patients (*10*,*13*–*15*). Few case studies reported successful treatment of patients with a solitary brain lesion or initiation of therapy before the infection entered the brain (*5*,*10*). The first successful treatment of AIDS-related GAE was reported in 2000 (*4*).

The condition of our patient deteriorated under empirical treatment with antimicrobial agents previously used to treat *Acanthamoeba* infection. When anti-amoebic therapy was changed to peroral and topical miltefosine, the skin lesions healed and the brain lesions regressed. After the remaining brain lesion had been surgically excised, the patient could be discharged from the hospital. Two years after treatment ended, the patient is partly rehabilitated with no signs of amoebic or mycobacterial infection.
